# Dehydration-associated cerebral hypoperfusion in sudden sensorineural hearing loss: an arterial spin labeling-based preliminary study

**DOI:** 10.3389/fneur.2025.1647804

**Published:** 2025-09-10

**Authors:** Palpasa Shrestha, Zhi Wen, Anyuan Zheng, Xilin Yang, Hua Liao, Mohamed Muntasir Ramjaun, Cixing You, Jati Shrestha, Renjie Yang, Jun Chen

**Affiliations:** ^1^Department of Radiology, Renmin Hospital of Wuhan University, Wuhan, China; ^2^Department of Otolaryngology-Head and Neck Surgery, Renmin Hospital of Wuhan University, Wuhan, China; ^3^Department of Neurosurgery, National Trauma Center, Kathmandu, Nepal

**Keywords:** arterial spin labeling, blood viscosity, cerebral blood flow, dehydration, sudden sensorineural hearing loss

## Abstract

**Objective:**

Sudden sensorineural hearing loss (SSNHL) rapidly decreases hearing, often by more than 30 decibels within 3 days. While circulatory issues are suspected causes, the exact reason remains unclear. This study employs Arterial Spin Labeling to examine changes in cerebral blood flow and its relationship with hydration levels in SSNHL.

**Methods:**

A prospective study examined patients with SSNHL, dividing them into 20 right-sided SSNHL (RSSNHL), 22 left-sided SSNHL (LSSNHL), and 20 healthy controls (HC). Cerebral blood flow (CBF) data were obtained using MATLAB. Statistical analysis included one-way ANOVA with *post hoc* analysis among RSSNHL, LSSNHL, HC, and Pearson correlation to explore the relationship with clinical variables.

**Results:**

Compared to HC, patients with RSSNHL showed reduced CBF value in right medial superior frontal gyrus, Heschl's gyrus (HG), and left inferior temporal gyrus, conversely increased perfusion in left calcarine. In LSSNHL patients, CBF value was decreased in the left superior temporal gyrus (STG) and right middle temporal gyrus, with increased perfusion in the left temporal pole STG compared to HC (cluster level *P* < 0.05 after FDR correction). Furthermore, CBF in the right HG of RSSNHL and left STG of LSSNHL negatively correlated with blood viscosity (*r* = −0.621, *P* = 0.003; *r* = −0.560, *P* = 0.007) and urine specific gravity (*r* = −0.483, *P* = 0.031; *r* = −0.485, *P* = 0.022), and positively correlated with daily water intake (*r* = 0.650, *P* = 0.002; *r* = 0.568, *P* = 0.006).

**Conclusion:**

Cerebral perfusion changes were present in the temporal, frontal, and occipital lobes of SSNHL patients. Furthermore, insufficient water intake may be a potential cause of SSNHL. Drinking adequate water is vital in preventing and recovering from this condition.

## Highlights

- Arterial spin labeling analysis to observe cerebral blood flow rate alteration in Sudden sensorineural hearing loss (SSNHL) patients.
- Addresses the possible pathophysiology mechanism of SSNHL.
- Highlights the importance of intake of an adequate amount of water.

## 1 Introduction

Sudden sensorineural hearing loss (SSNHL) is a distressing condition often considered an otologic emergency due to its rapid onset (1). It is defined as a hearing loss of 30 dB or more for three consecutive frequencies that appears within 3 days and is accompanied by tinnitus, vertigo, and other discomforts. SSNHL is mostly unilateral (1, 2). The prevalence in adults is about 5–20 cases per 100,000 persons (2). Importantly, in 90% of SSNHL cases, despite adequate examination, the pathophysiology remains unclear, highlighting the complexity of this disease to understand (1, 3). Over the years, several potential hypotheses have been put forth, including vascular disorders (microthrombosis, vasospasm, hyperviscosity), infections (cytomegalovirus, meningitis, fungal meningitis, AIDS, mumps, syphilis), autoimmune disease (systemic lupus erythematosus, Behçet's disease, Cogan's syndrome), neural (retrochoclear neurodegeneration), and membrane rupture (2, 4, 5). The cause of idiopathic SSNHL and the proper treatment of its patients are still up for debate despite a great deal of research (3, 6). Even though many theories about the pathophysiology of SSNHL have been presented, the vascular theory is the most recognized (3). It is well-recognized that vascular risk factors can damage the vascular endothelium, leading to functional impairments in the inner ear (7). Based on vascular etiologic theory, acute vascular hemorrhage, vascular disease, vasospasm, or a change in blood viscosity (BV) could cause SSNHL (3, 5). Some research shows that BV is inversely related to blood flow (5), and elevated BV results in greater blood flow resistance, reducing cerebral blood flow (CBF) (8). Previous studies have revealed the vital role of measuring BV in patients with SSNHL, as changes in microcirculation may be involved in the etiology and correlating whole blood viscosity to decreased CBF (8, 9).

Furthermore, BV functions as an independent marker for the hydration status of individuals (10), and dehydration adversely affects both systemic and regional blood flow (11). Some research showed that dehydration affects the cardiovascular system, resulting in vascular insufficiency (10, 12), and has a detrimental effect on the frequency, severity, and prognosis of SSNHL (12). Previous research states that dehydration affects cognitive performance and indicates the importance of increasing fluid intake (13). In addition to hearing loss, an individual's quality of life is negatively impacted by a variety of non-auditory issues, including social isolation, depression, anxiety, and dementia (14). Asymmetrical hearing in patients with sudden sensorineural hearing loss (SSNHL) often causes localization problems, which can be irritating and even distressing for the affected individual. The prognosis concerning hearing recovery depends on various factors, including age at onset, degree of hearing loss, severity of vertigo, and time from onset to treatment visit (3). Therefore, these complexities highlight the ongoing need for research to determine contributing factors.

Recent advancements in imaging techniques, such as Arterial Spin Labeling (ASL), have enabled CBF measurements, offering new insights into changes in cerebral perfusion. ASL evaluates brain perfusion through magnetic resonance imaging (MRI), and its key advantage is that it does not require an external tracker, such as gadolinium. The labeled water molecules can move beyond the intravascular space (15). Compared to PET or contrast-enhanced MRI, ASL is non-invasive and produces quantitative CBF maps with excellent repeatability. Due to its sensitivity to changes in microvascular flow, ASL is especially good at detecting minute changes in perfusion inside the auditory cortices, such as the HG and STG (16).

In an fMRI study, SSNHL patients showed altered connections in the salience, default mode, and central executive networks, with decreased functional connectivity between the insula and other brain regions (14). A functional connectivity fMRI study highlighted that the reorganization of the anterior cingulate cortex and the disruption of multiple intrinsic networks are crucial in regulating cognitive, emotional, and auditory function processing in SSNHL patients (17). Furthermore, a study investigating the topological alterations within the complete brain network of SSNHL patients indicated modifications present in the white matter network (16).

Most SSNHL research primarily focuses on topographical changes, structural alterations, particular regions of interest (ROIs), and functional brain connectivity networks, often neglecting possible perfusion changes that could clarify the pathophysiological processes involved. Although the theory of vascular compromise is well-established, limited studies examine how systematic hemodynamic changes influence SSNHL. Furthermore, despite the growing use of ASL in auditory research, the effects of SSNHL on cerebral perfusion and its underlying mechanisms are still poorly understood. ASL studies provide important insights into the mechanisms of vascular compromise and focus on cerebral perfusion (18). Our study fills the gap left by previous research, which ignores the importance of hydration. This discrepancy is essential since the brain-auditory axis is sensitive to changes in perfusion, and the cochlea requires sufficient CBF. By analyzing the variations in CBF values among SSNHL patients and investigating the possible contribution of dehydration to these fluctuations, this study sought to fill this information gap.

This research uses ASL imaging and a detailed analysis of 170 brain regions to compare and address the alteration in CBF value in SSNHL patients to healthy controls. The study aims to document the alterations in brain areas and identify the connection between hydration and SSNHL pathophysiology. Correlation analysis with the clinical data of the patients seeks to explore the differential mechanism of brain intrinsic function changes in patients with SSNHL.

## 2 Materials and methods

### 2.1 Subjects

**Figure 1:**
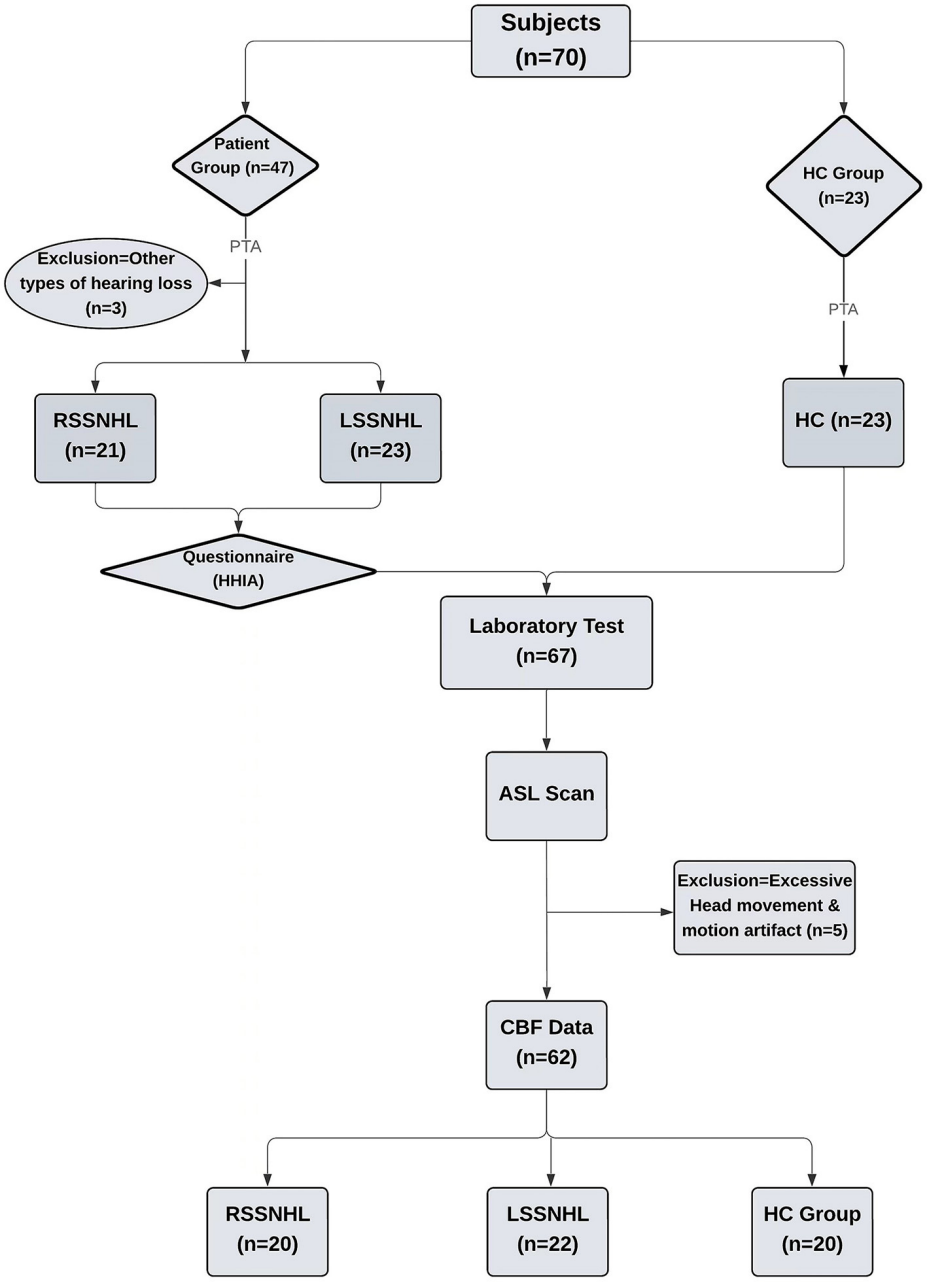
Flowchart of subject recruitment.

This prospective study enrolled SSNHL patients admitted to the otorhinolaryngology department of Renmin Hospital of Wuhan University from August 2023 to June 2024. Participants included 23 healthy controls (HC) and 47 hearing loss patients. Following the Pure Tone Average (PTA) test, three patients were excluded due to other types of hearing loss. Based on the PTA results, 44 patients were divided into right-sided Sudden Sensorineural Hearing Loss (RSSNHL) and left-sided Sudden Sensorineural Hearing Loss (LSSNHL). The PTA test was administered to the HC group to ensure study standardization. Each SSNHL patient completed the Hearing Handicap Inventory for Adults (HHIA) questionnaire. An ASL scan was performed after laboratory testing for all participants, including SSNHL and HC groups. Following the exclusion of five subjects due to excessive head movement and motion artifacts, 42 SSNHL patients (19 male and 23 female, aged 19–70 years) and 20 healthy controls (10 male and 10 female, aged 19–68 years) were included in the final analysis. These groups were matched for age, gender, and educational level. Detailed information on the recruitment process and all the included steps are given in the flowchart (Figure 1). The inclusion criteria were as follows: 1) Acute hearing loss with no apparent cause, 2) hearing loss above 30 dB in three sequential frequencies, 3) symptom onset and diagnosis within 1 to 7 days, 4) age < 75 years (1, 5), and 5) no prior treatment (e.g., steroids).

Exclusion criteria included: 1) mixed hearing loss or Bilateral hearing loss, 2) history of sudden deafness, 3) ear disorders (intracochlear hemorrhage, Schwannoma, Meniere disease, or other infectious diseases), 4) claustrophobia or contraindication to MRI, 5) pregnancy, 6) prior ear surgery or brain trauma, and 7) disease duration >7 days were not included.

The Renmin Hospital of Wuhan University Medical Ethics Committee approved this study (WDRY2024-K275), following the principles of the Declaration of Helsinki.

### 2.2 Audiometry test

The Pure-Tone Average (PTA) was calculated to evaluate hearing thresholds through pure-tone audiometry. An otolaryngologist from our hospital performed the PTA across frequencies ranging from 0.125 to 8 kHz. The average PTA for SSNHL patients exceeded 30 dB HL across all frequencies, while the healthy control group had an average PTA of < 20 dB HL. Based on these PTA results, SSNHL patients were categorized into two subgroups: RSSNHL (*n* = 20) and LSSNHL (*n* = 22) (Figure 1).

### 2.3 Clinical assessment

Demographics, including age, gender, education level, handedness, related symptoms, and lifestyle factors (e.g., smoking and alcohol consumption), were collected from all SSNHL and HC groups. Clinical data such as disease duration and audiometry test for SSNHL patients. Laboratory tests were conducted to measure biochemical variables, including urine-specific gravity (U_sg_) and blood viscosity (BV) for both participants. The Hearing Handicap Inventory for Adults (HHIA) questionnaire was administered to assess the social and emotional impacts of hearing loss on daily life. The questionnaire consists of 25 items, with response scores of 0 for “No,” 2 for “Sometimes,” and 4 for “Yes.” Total scores were categorized as follows: 0–16 (No Handicap), 18–42 (Mild to Moderate Handicap), and 44 or above (Significant Handicap) (19).

### 2.4 Hydration assessment

U_sg_ is a sensitive indicator of hydration status, with a standard reference range of 1.003–1.030 μmol/L (21). Due to its simplicity, speed, and affordability, U_sg_ is a popular alternative for determining participants' hydration levels in clinical settings (20). U_sg_ indicates that the patient's hydration level correlates with urine osmolality and illustrates the kidneys' capacity for concentration (21). All participants were instructed to continue fasting and refrain from drinking water before collecting urine and blood samples. Random morning samples (midstream urine) were taken from each participant during the baseline medical assessment. Blood Viscosity (BV), measured at different shear rates ranging from 0.1 s^−1^ to 1,000 s^−1^ (22), was also considered a biomarker of an individual's hydration status (10). BV was measured at various shear rates (low shear, medium shear, high shear) (23). Additionally, participants were asked questions regarding daily water consumption. The recommended average daily intake is 3.0 L for men and 2.5 L for women (24).

### 2.5 MRI acquisition

MRI scans were performed using a 3.0 Tesla MRI scanner (Signa Architect Air GE Healthcare, USA) with a 48-channel phased array head coil. The participants were given rubber-soft foam and earplugs to reduce equipment noise. ASL parameters Repetition time (TR) = 4,854 ms; echo time (TE) = 10.7 ms; slice thickness = 4 mm; FOV = 240 mm × 240 mm, Post label delay (PLD)= 2,025 ms; flip angle = 111; Labeling duration = 1,800 ms; Labeling offset: 90 mm; Readout method: 3D fast spin-echo and slice number = 36. All participants had MRI scans while keeping their eyes closed.

### 2.6 Data processing

**Figure 2:**
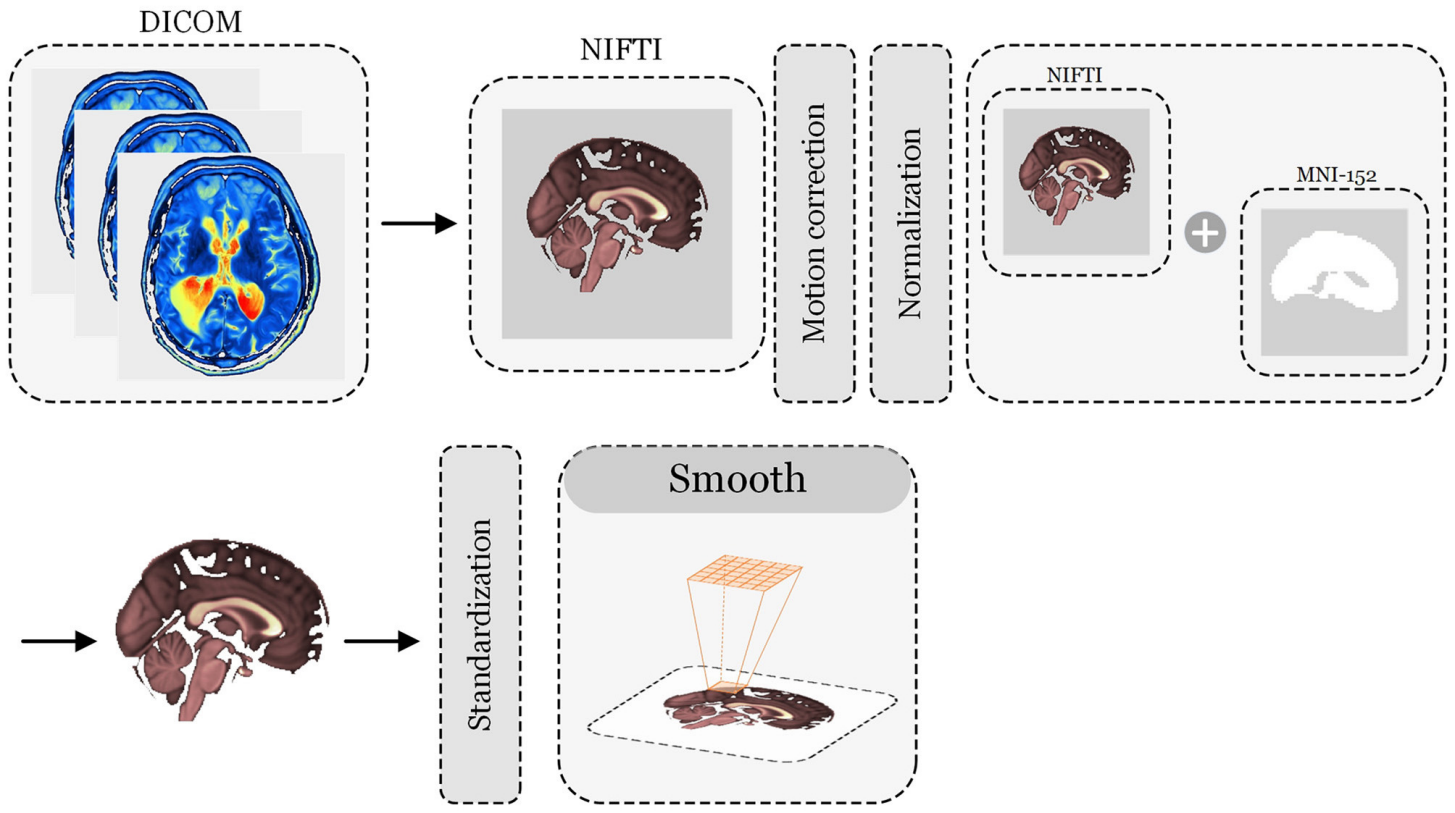
Data processing steps.

ASL data were collected using a GE MR scanner and processed using MATLAB software per established procedures (15) (Figure 2). The data processing steps included:

a.Converted 3D ASL DICOM files to NIfTI format, applied head motion correction, and co-registered T1 images with ASL data to ensure precise spatial normalization to Montreal Neurological Institute brain templates (MNI-152). b. Matlab-based Statistical Parametric Mapping (SPM12) was used to standardize the CBF value of each voxel, which was obtained by subtracting the mean CBF and dividing it by the standard deviation. The CBF value was smoothed with a Gaussian Kernel of 6 × 6 × 6 mm^3^. Anatomical Labeling (AAL3v1) atlas (25), based on MATLAB software, was used to segment and obtain normalized CBF value, followed by a one-way ANOVA to compare the three groups (RSSNHL, LSSNHL, HC), corrected using the False discovery rate (FDR)method, and followed by *post hoc* analyses, e. Overlaying and visualizing the results using AAL3v1 and Xjview.

### 2.7 Analysis of CBF in auditory and non-auditory brain regions

Based on previous studies indicating the primary auditory cortex as a central location for sound processing, the temporal lobe was selected as the ROI for analyzing both auditory and non-auditory brain regions (26). The AAL3V1 atlas further divided the temporal lobe into subregions as Heschl's gyrus (HG), superior temporal gyrus (STG), temporal pole superior temporal gyrus (TP-STG), middle temporal gyrus (MTG), temporal pole middle temporal gyrus (TP-MTG), and inferior temporal gyrus (ITG) (25). Since HG and STG are the primary auditory cortex, ROI analysis was focused on these areas for further Pearson correlation analysis.

### 2.8 Statistical analysis

Statistical analyses were conducted using MATLAB and SPSS. A one-way ANOVA test was used to compare the mean and standard deviation of the demographic and clinical data, as well as to compare the CBF values between the three groups: RSSNHL patients, LSSNHL patients, and the HC group using the False Discovery Rate (FDR) method. It was used to correct for multiple comparisons with a significance level of voxel-level *P* < 0.001 and cluster-level *P* < 0.05, followed by *post hoc* pairwise comparisons. The continuous variable obeys a normal distribution. The chi-square test was used for categorical data. A *P*-value < 0.05 was considered significant. Pearson correlation was used to assess the strength and correlation between perfusion change and clinical variables, with *P* < 0.05 considered significant. Linear regression was used to model the relationship between the variables further.

## 3 Results

### 3.1 Demographic and clinical data of SSNHL patients and HC

No significant differences were observed in age, gender, education level, handedness, hearing duration, PTA, and HHIA score between SSNHL patients and the HC group (Table 1). However, a significant difference was found in U_sg_ and daily water consumption between these groups. All participants had normal kidney function test results.

**Table 1:** Demographic and clinical characteristics.

**Characteristics**	**RSSNHL (*n* = 20)**	**LSSNHL (*n* = 22)**	**HC (*n* = 20)**	***P*-value**
Age (years)	39.10 ± 13.14	37.61 ± 11.63	34.20 ± 10.05	0.542
Gender (M/F)	9 M/11 F	10 M/12 F	10 M/10 F	0.7
Education level (years)	11.17 ± 1.82	11.60 ± 1.72	10.95 ± 1.43	0.261
Handedness (R/L)	20/0	22/0	20/0	-
Duration of hearing loss (Day)	2.55 ± 1.2	2.12 ± 08	-	-
HHIA score	31.7 ± 10.27	30.5 ± 10.98	-	-
PTA of affected ear (dB)	58.28 ± 23.6	57.33 ± 13.9	-	-
BV (low shear)	21.56 ± 2.16	21.67 ± 2.25	19.32 ± 1.26	< 0.001
BV (medium shear)	5.28 ± 0.80	4.91 ± 0.49	4.58 ± 0.41	0.002
BV (high shear)	4.36 ± 0.71	4.29 ± 0.65	3.81 ± 0.42	0.011
U_sg_	1.026 ± 0.007	1.027 ± 0.007	1.011 ± 0.002	< 0.001
Water intake per day (ml)	1,090.48 ± 634	1,109.09 ± 516	2,395 ± 414	< 0.001

Values are measured in mean ± SD. SSNHL, Sudden Sensorineural Hearing Loss; HC, Healthy Control; HHIA, Hearing Handicap Inventory for Adults; PTA, Pure tone average; U_sg_, Urine-specific gravity.

### 3.2 Cerebral perfusion comparison between three groups (LSSNHL vs. RSSNHL vs. HC)

One-way ANOVA with FDR correction, followed by *post hoc* analyses, revealed significant differences in cerebral perfusion across three groups.

**Figure 3:**
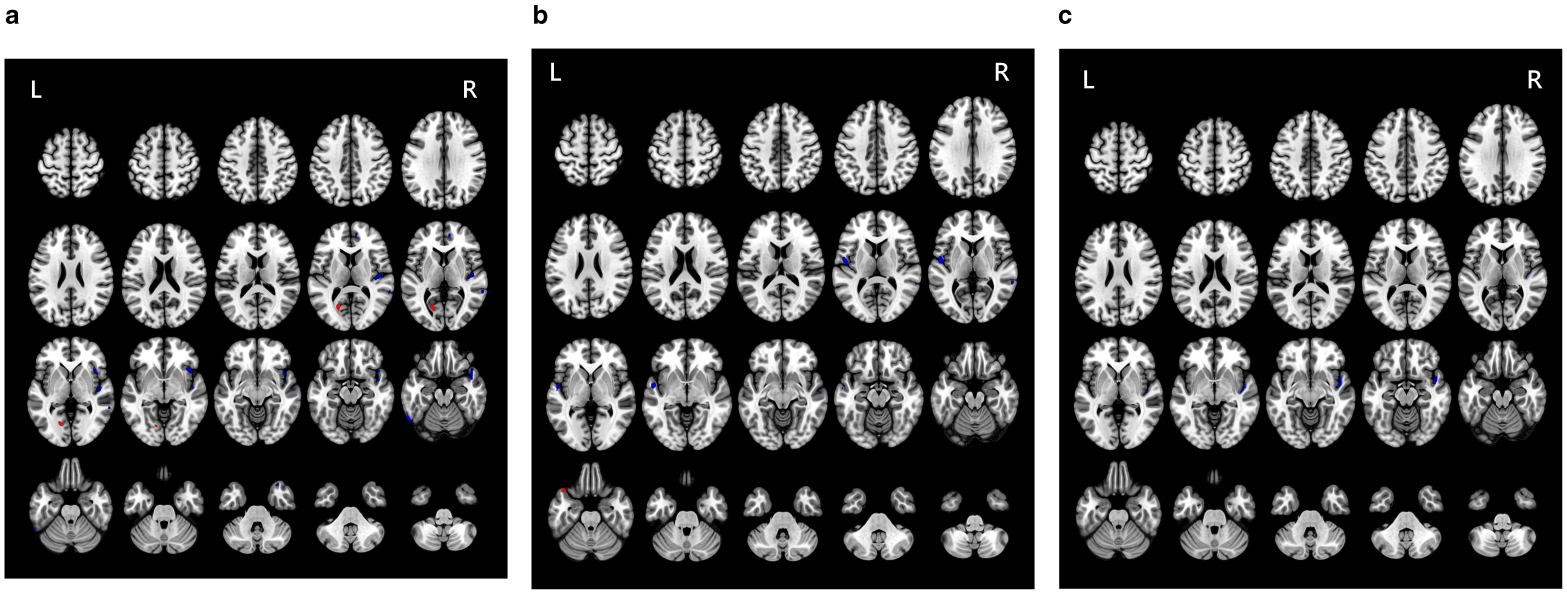
**(a**) Alteration of perfusion in different brain regions between the RSSNHL and the HC group. Red indicates the brain areas with increased blood flow, and blue indicates those with decreased blood flow. **(b)** Alteration of perfusion in different brain regions between the LSSNHL and the HC group. Red indicates the brain areas with increased blood flow, and blue indicates those with decreased blood flow. **(c)** Alteration of perfusion in different brain regions between the RSSNHL and the LSSNHL group. Blue indicates those with decreased blood flow.

Compared to HC, RSSNHL patients exhibited decreased CBF values in the right medial superior frontal gyrus (SFG), insula, HG, MTG, and left ITG, alongside increased perfusion in the left calcarine (Figure 3a, Table 2). The LSSHNL patients showed decreased CBF values in the left STG, left Rolandic operculum, and right MTG, and increased perfusion in the left TPSTG compared to the HC group (Figure 3b, Table 2). Compared to RSSNHL, LSSNHL patients had significantly decreased CBF values in the right STG (Figure 3c, Table 2).

**Table 2:** Brain regions with statistically significant clusters of CBF between three groups (LSSNHL vs. RSSNHL vs. HC).

**Condition (CBF value)**	**Brain region**	**MNI coordinates (x,y,z)**	**Peak T score**	**Cluster size (voxels)**
RSSNHL < HC	R-SFG (medial)	(10,50,6)	−4.5	75
	R-HG	(46,−16,5)	−5.3	564
	R-Insula	(40,17,−5)	−3.37	380
	R-MTG	(62,−42,5)	−5.7	90
	L-ITG	(−58,−60,−20)	−2.9	150
RSSHHL>HC	L-Calcarine	(−20,−66,8)	4.2	390
LSSNHL < HC	L-STG	(−52,−2,−5)	−3.32	332
	L-Rolandic operculum	(−50,−7,10)	−4.34	300
	R-MTG	(64,−42,6)	−4.2	70
LSSNHL>HC	L-TPSTG	(−44,22,−22)	5.58	80
LSSNHL < RSSNHL	R-STG	(46,−4,−10)	−5.4	434

P < 0.05 was considered statistically significant after FDR correction.

CBF, Cerebral blood flow; SSNHL, Sudden Sensorineural Hearing loss; LSSNHL, Left sided Sudden Sensorineural Hearing loss; RSSNHL, Right sided Sudden Sensorineural Hearing loss; L, Left; R, Right; MNI, Montreal Neurologic Institute; SFG, Superior frontal gyrus; HG, Heschl's gyrus; MTG, Middle temporal gyrus; ITG, Inferior temporal gyrus; STG, Superior temporal gyrus; TP STG, Temporal pole superior temporal gyrus.

### 3.3 Pearson correlation analysis

The Pearson correlation analysis revealed distinct patterns of association between cerebral blood flow (CBF) in key auditory regions and hydration-related parameters among SSNHL subgroups.

**Figure 4:**
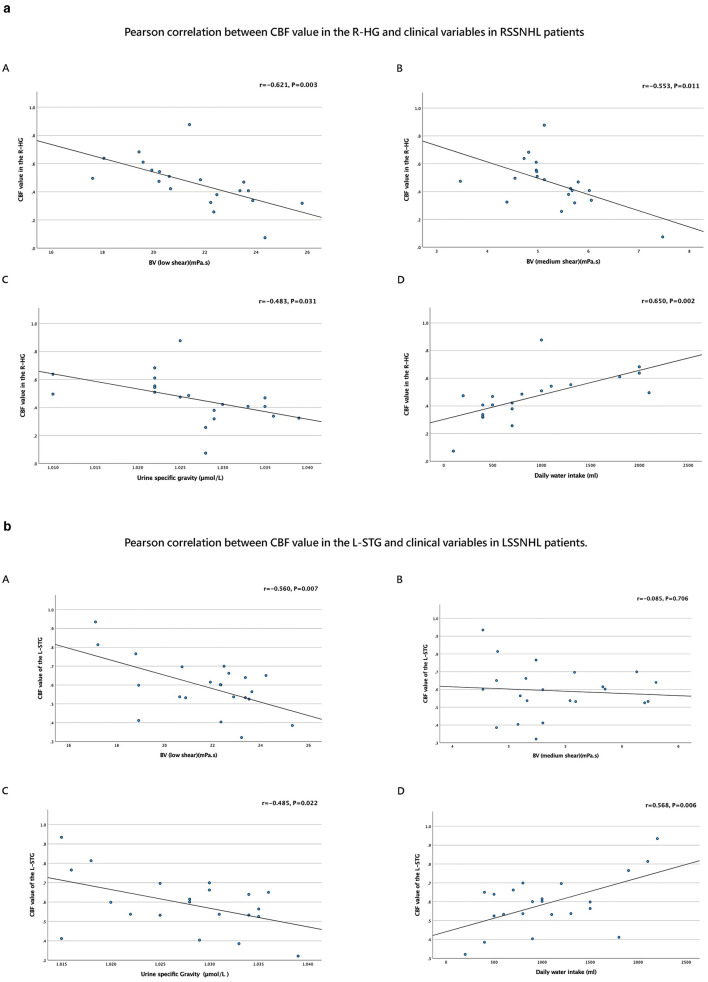
**(a)** Pearson correlation between the CBF value of R-HG and clinical variables in SSNHL. Pearson correlation analysis between (A) CBF value of R-HG and BV (low shear), (B) CBF value of R-HG and BV (medium shear), (C) CBF value of R-HG and Urine specific gravity, (D) CBF value of R-HG and Daily water intake (ml). **(b)** Pearson correlation between the CBF value of L-STG and clinical variables in LSSNHL. Pearson correlation analysis between (A) CBF value of L-STG and BV (low shea), (B) CBF value of L-STG and BV (medium shear), (C) CBF value of L-STG and Urine specific gravity, (D) CBF value of L-STG and Daily water intake (ml).

In RSSNHL patients, the right HG's CBF value was negatively correlated with BV at low and medium shear rates (mPa.s) and with U_sg_ (μmol/L). Additionally, it showed a positive correlation with daily water intake (ml; Table 3, Figure 4a).

**Table 3:** Pearson correlation between SSNHL patients and clinical variables.

**Group**	**Brain regions**	**BV (low shear)**	**BV (medium shear)**	**Urine specific gravity**	**Daily water intake (ml)**
RSSNHL	R-HG	*r* = −0.621, *P* = 0.003	*r* = −0.553, *P* = 0.011	*r* = −0.483, *P* = 0.031	*r* = 0.650, *P* = 0.002
LSSNHL	L-STG	*r* = −0.560, *P* = 0.007	*r* = −0.085, *P* = 0.706	*r* = −0.485, *P* = 0.022	*r* = 0.568, *P* = 0.006

In LSSNHL patients, the left STG's CBF value showed a negative correlation with BV (low shear; mPa.s) and U_sg_ (μmol/L). Conversely, it positively correlated with daily water consumption (ml; Table 3, Figure 4b).

No significant correlation was found between the CBF of healthy controls and clinical variables (all *P* > 0.05).

Also, no significant correlation was observed between the Hearing Handicap Inventory for Adults (HHIA) score and altered perfusion in these brain regions. Additionally, no significant correlation was observed between other brain regions and HHIA (all *P* > 0.05).

### 3.4 Linear regression analysis

In RSSNHL patients, R-HG showed a significant relationship between BV low shear, U_sg_, Daily water intake, Hematocrit, and PTA. In LSSNHL patients, L-STG also showed a substantial relationship between BV low shear, U_sg_, Daily water intake, Hematocrit and PTA, respectively (Table 4).

**Table 4:** Linear regression between SSNHL patients and clinical variables.

**Brain region**	**Predictor**	**β (SE)**	**95% CI**	***R* ^2^**	**F statistic**	***P*-value**
R-HG	BV (Low shear)	−0.0490 (0.015)	[−0.080, −0.018]	0.386	11.29	0.003
	Urine specific gravity	−10.7987 (4.608)	[−20.480, −1.117]	0.23	5.49	0.03
	Daily water intake (mL)	0.0002 (4.88e-5)	[7.47e-5, 0.0001]	0.423	13.18	0.001
	Hematocrit (Hct)	−0.1189 (0.031)	[−0.185, −0.053]	0.445	14.44	0.001
	PTA (dB)	−0.0086 (0.002)	[−0.012, −0.005]	0.596	26.53	0.0001
L-STG	BV (Low shear)	−0.0357 (0.012)	[−0.060, −0.011]	0.314	9.147	0.006
	Urine specific gravity	−9.6422 (3.890)	[−17.757, −1.527]	0.235	6.143	0.022
	Daily water intake (mL)	0.0001 (4.62e-5)	[4.63e-5, 0.0002]	0.323	9.535	0.005
	Hematocrit (Hct)	−0.0847 (0.020)	[−0.125, −0.044]	0.484	18.78	0.0003
	PTA (dB)	−0.0057 (0.002)	[−0.009, −0.002]	0.352	10.86	0.003

## 4 Discussion

Water serves as the essence of life and constitutes the primary element in cells, tissues, and organs. Different organs vary in water content, with 83.0% found in blood, 74.8% in the brain, and 22.0% in the skeletal muscle system (27). This study aimed to investigate alterations in cerebral perfusion in auditory and non-auditory brain regions in individuals with SSNHL and evaluate how these changes relate to hydration. Prior research has not extensively explored this concept. One of the unique features of this study is its focus on the link between alterations in CBF value and hydration parameters in patients with SSNHL.

The primary auditory cortex (PAC) comprises Heschl's gyrus and the superior temporal gyrus (STG), both of which are essential for auditory processing. Anatomically, Heschl's gyrus—often the central auditory area—corresponds to Brodmann areas 41 and 42. Its primary function is the early processing of auditory information, such as sound intensity and frequency, and it is situated in the transverse temporal gyrus (28). Furthermore, the importance of the STG in auditory health has been highlighted by volumetric studies that have linked changes in the STG to several auditory diseases, such as tinnitus and hearing loss (29). These regions are responsible for higher-level auditory data processing, which leads to auditory perception (30, 31). This study found decreased CBF value in the affected side of SSNHL patients, specifically in the HG and STG regions integral to auditory processing. These findings align with other fMRI studies (32, 33), which suggest that alterations in the auditory cortex may disrupt the processing and timing of incoming auditory information (26, 34). Additionally, gray and white matter changes in the auditory cortex have been observed in voxel-based morphometry (VBM) and diffusion tensor imaging (DTI) studies, pointing to a reduced executive control network in individuals with persistent hearing loss (35). Reduced perfusion in HG and STG may thus impair SSNHL patients' ability to process and interpret sound, leading to difficulties in speech comprehension, sound localization, and understanding spoken words (33).

The middle and inferior temporal gyrus plays a pivotal role in supporting visual perception, multimodal sensory integration, and the processing of linguistic and semantic memory (36). Gray matter volume decrease in MTG and ITG was reported in an fMRI investigation on brain structural and functional changes in people with unilateral SSNHL (37). Decreased CBF value in MTG and ITG was also found in this study, which might have affected their multimodal functions. The default mode network has been suggested to include the medial region of the superior frontal gyrus (SFG), which is frequently deactivated during cognitively relevant processing (38). Previous studies have identified the SFG as an essential component of the auditory processing network, integrating a wide range of sensory inputs and coordinating central nervous system activity (39). Decreased perfusion in this region may impair auditory processing and cognitive function in SSNHL patients. Another study of SFG, which plays a vital role in linguistic cognition (40), suggests that patients with SSNHL may have decreased functional connectivity in a SFG, and language memory might be impacted. Notably, the reduced perfusion in the STG and SFG, a critical area for multisensory integration, may help explain the non-auditory symptoms of SSNHL, including tinnitus (41).

This study's results are consistent with these findings, showing a decreased CBF, which may indicate a disruption in multimodal cognitive processes. In addition, a functional connectivity study that assessed the functional connectivity of brain networks in patients with SSNHL demonstrated that changes in connectivity are related to changes in auditory and cognitive function (32).

The insula, which separates the temporal lobe from the frontal and parietal lobes, plays a vital role in audio-visual and sensory integration and emotional processing. Previous research has shown hyperperfusion and decreased functional connectivity in the insula of SSNHL patients (14). However, this study observed reduced perfusion in the insula, which may suggest different pathophysiological mechanisms at play. The rostral temporal pole within the meso-temporal lobe is associated with higher cognitive functions such as emotion, social cognition, autobiographical memory, and multimodal sensory integration (42). Conversely, enhanced blood flow in the visual areas, notably the calcarine region, indicates the presence of cross-modal plasticity, which may compensate for auditory impairments. This study revealed hyperperfusion in the TP STG and calcarine regions, potentially indicating a compensation mechanism. The temporal pole, superior temporal gyrus, and calcarine areas may have higher CBF due to increased neuroplasticity. Patients with hearing loss can benefit from compensatory auditory processing in the temporal regions and cross-modal plasticity in the calcarine cortex, where the brain increases its dependence on visual information to compensate for auditory impairments.

This study found that the CBF value in the right HG in RSSNHL patients and the left STG in LSSNHL patients was negatively correlated with BV(low shear) and U_sg_. In RSSNHL patients, R-HG also showed a negative correlation with BV(medium shear). Furthermore, the left STG in LSSNHL patients and the right HG in RSSNHL patients positively correlated with daily water consumption. These findings suggest that increased BV, U_sg_, and decreased water intake may be associated with reduced CBF value in this critical auditory region. A few studies suggested that abnormalities in CBF in SSNHL are secondary to vascular anomalies and that impaired microvascular flow dynamics can significantly influence auditory processing (5). The results of our study support the “vascular compromise” theory, which postulates that dehydration (10) and BV hinder cochlear blood flow by causing microcirculatory sludging (9) extending to auditory cortices. This aligns with the study indicating that BV increases precede hearing loss (43). Changes in blood vessel tone and blood volume can result in congestion, lack of oxygen, and increased capillary permeability, all of which can negatively affect auditory processing (43). The results support this hypothesis, stating that higher BV appears to influence microvascular dynamics and immune responses, adversely affecting auditory processing.

Furthermore, the inverse relationship between hydration, as reflected by BV, U_sg_, fluid intake, and CBF, emphasizes the role of appropriate hydration in maintaining adequate cerebral perfusion. The BV-CBF association indicates that the “vascular steal” phenomenon, in which dehydration diverts blood from auditory circuits, is modulated by hydration (11). This study emphasizes the significant relationship between hydration status and perfusion in auditory brain regions, suggesting dehydration may contribute to impaired blood flow and auditory dysfunction in SSNHL patients. BV could also serve as a potential marker for recovery in SSNHL (9, 43), as dehydration status could be defined by BV and U_sg_ (10, 24, 44). We can also establish that U_sg_ is sensitive to changes in hydration (44). Our study is consistent with previous studies on water intake, emphasizing that dehydration leads to reduced cerebral blood flow and impaired cognitive function, as well as the importance of maintaining an appropriate daily water intake strategy and maintaining proper fluid intake to avoid physiological problems associated with insufficient body hydration (11). Linear regression analysis also provided further support for these results.

Recent brain imaging studies have also shown how dehydration affects blood vessel changes and cognitive performance, suggesting a possible treatment target for patients with SSNHL (13, 45). Additionally, clinical studies have proven that hydration status significantly impacts the outcome of SSNHL, highlighting the importance of maintaining adequate fluid intake to prevent dehydration-related physiological problems (12). This study shows that hydration status affects the prognosis and also the prevention of SSNHL.

## 5 Limitation

The main limitation of this study is its relatively small sample size, which diminishes the statistical power of the findings. A larger sample could yield more definitive results and allow for the identification of subtle differences in CBF among groups. Additionally, the cross-sectional design of this study restricts our ability to establish causal relationships. Although the study explored the connection between cerebral perfusion, hydration, and hearing loss, further longitudinal studies are needed to better understand the timeline and lasting effects of sudden sensorineural hearing loss (SSNHL) on brain functions and hydration levels. Ultimately, although this study reveals connections between dehydration and alterations in cerebral perfusion, further research is needed to investigate how dehydration affects neurotransmitter activity and cognitive processing in SSNHL patients. This will enhance our understanding of the specific mechanisms by which dehydration influences both auditory and mental functions. Our study does not prescribe a specific hydration protocol; however, ensuring sufficient water intake, screening SSNHL patients for dehydration, and promoting fluid consumption may serve as effective components of a hydration strategy.

## 6 Conclusion

This study provides new insights into cerebral perfusion changes in auditory and non-auditory brain regions of patients diagnosed with SSNHL. Reduced blood flow was observed in critical areas associated with auditory, cognitive, and emotional processing, as well as sensory compensation mechanisms, following hearing loss. These findings highlight the crucial role of adequate hydration in enhancing brain perfusion, which may facilitate recovery in patients with SSNHL. The study emphasizes the need for a deeper understanding of hydration's impact and potential benefits in the therapeutic management of SSNHL, extending beyond merely addressing symptoms. Further research is needed to investigate hydration strategies as part of a comprehensive treatment approach for SSNHL.

## Data Availability

The raw data supporting the conclusions of this article will be made available by the authors, without undue reservation.
