# Supplementary material for: Dehydration-associated cerebral hypoperfusion in sudden sensorineural hearing loss: an arterial spin labeling-based preliminary study
Source: Front Neurol. 2025 Sep 10;16:1647804. doi: 10.3389/fneur.2025.1647804 (PMC12457104; doi:10.3389/fneur.2025.1647804)
Supplement: Supplementary file 1 [file Table_1.docx]

**Supplementary Materials**

**Table S1: List of abbreviations**

| **Abbreviation** | **Full Name** | **Abbreviation** | **Full Name** |
| --- | --- | --- | --- |
| CBF | Cerebral blood flow | HG | Heschl’s gyrus |
| SSNHL | Sensorineural Hearing loss | STG | Superior temporal gyrus |
| HCT | Hemocrit | PAC | Primary auditory cortex |
| HC | Healthy controls | AAL3v1 | Automated Anatomical Labeling atlas, version 3, release 1 |
| BV | Blood Viscosity | TP STG | Temporal pole superior temporal gyrus |
| MTG | Middle temporal gyrus | SFG | Superior frontal gyrus |
| ITG | Inferior temporal gyrus | U_sg_ | Urine specific gravity |

**Table S2: HHIA questionnaire and scoring**

The purpose of this scale is to help identify the possible distress that hearing loss may cause to patients;

Please answer each question individually; yes: 4 points; sometimes: 2 points; no: 0 points.

| S. No. | **Questions** | **Yes** | **No** | **Sometimes** |
| --- | --- | --- | --- | --- |
| 1 | Does your hearing problem make you use the phone less often that you would like? |  |  |  |
| 2 | Does your hearing problem make you feel embarrassed or out of place when you are meeting new people? |  |  |  |
| 3 | Does your hearing problem make you avoid groups of people? |  |  |  |
| 4 | Does your hearing problem make you irritable? |  |  |  |
| 5 | Does your hearing problem make you feel frustrated or unhappy when talking to family members? |  |  |  |
| 6 | Does your hearing problem cause difficulties when you go to a party or social gathering? |  |  |  |
| 7 | Does your hearing problem make you frustrated when talking to your  coworkers, clients, or customers? |  |  |  |
| 8 | Do you experience hearing problems when you go to the movies or the  theater? |  |  |  |
| 9 | Do you feel handicapped by your hearing problem? |  |  |  |
| 10 | Does your hearing problem cause difficulties when visiting friends, relatives, or neighbors? |  |  |  |
| 11 | Does your hearing problem cause difficulties when talking to your coworkers, clients, or customers |  |  |  |
| 12 | Does your hearing problem make you nervous? |  |  |  |
| 13 | Does your hearing problem cause you to visit your friends, relatives, or  neighbors less than you would like? |  |  |  |
| 14 | Does your hearing problem cause you to have arguments of fights with your  family? |  |  |  |
| 15 | Does your hearing problem make it difficult to listen to the TV or radio? |  |  |  |
| 16 | Does your hearing problem make you go out shopping less than you would like? |  |  |  |
| 17 | Does your hearing problem make you feel alone or isolated? |  |  |  |
| 18 | Does your hearing problem make you feel annoyed or unhappy? |  |  |  |
| 19 | Does your hearing problem make you want to talk to family members less? |  |  |  |
| 20 | Do you believe your hearing problem reduces or limits you personal and / or social life? |  |  |  |
| 21 | Does your hearing problem cause difficulties when you go to a restaurant? |  |  |  |
| 22 | Does your hearing problem make you feel sad or depressed? |  |  |  |
| 23 | Does your hearing problem cause you to watch TV or listen to the radio less  than you would like? |  |  |  |
| 24 | Does your hearing problem make you feel embarrassed or uncomfortable when you talk to friends? |  |  |  |
| 25 | Does your hearing problem make you feel left out when you are with a group of people? |  |  |  |

**Table S3: Pearson correlation between Clusters found in RSSNHL and clinical variables**

| **Brain Region** |  | **BV (low shear) (mPa·s)** | **BV (medium shear) (mPa·s)** | **BV (high shear) (mPa·s)** | **Urine specific gravity (µmol/L )** | **Daily water intake (ml)** | **HHIA** |
| --- | --- | --- | --- | --- | --- | --- | --- |
| CBF value in the R-HG | Pearson Correlation | -0.621** | -0.553* | 0.013 | -0.483* | 0.650** | -0.147 |
|  | Sig. (2-tailed) | 0.003 | 0.011 | 0.958 | 0.031 | 0.002 | 0.536 |
|  | N | 20 | 20 | 20 | 20 | 20 | 20 |
| CBF value in the R-MTG | Pearson Correlation | -0.288 | -0.359 | -0.081 | -0.063 | 0.107 | 0.325 |
|  | Sig. (2-tailed) | 0.217 | 0.12 | 0.735 | 0.79 | 0.652 | 0.162 |
|  | N | 20 | 20 | 20 | 20 | 20 | 20 |
| CBF value in the L-ITG | Pearson Correlation | 0.252 | 0.241 | 0.202 | 0.227 | -0.326 | 0.093 |
|  | Sig. (2-tailed) | 0.285 | 0.305 | 0.394 | 0.335 | 0.16 | 0.697 |
|  | N | 20 | 20 | 20 | 20 | 20 | 20 |
| CBF value in the R-SFG(medial) | Pearson Correlation | -0.092 | -0.319 | 0.202 | -0.021 | 0.046 | -0.049 |
|  | Sig. (2-tailed) | 0.701 | 0.171 | 0.393 | 0.93 | 0.846 | 0.837 |
|  | N | 20 | 20 | 20 | 20 | 20 | 20 |
| CBF value in the R-Insula | Pearson Correlation | -0.011 | 0.181 | 0.028 | 0.003 | 0.162 | -0.351 |
|  | Sig. (2-tailed) | 0.964 | 0.446 | 0.908 | 0.99 | 0.494 | 0.129 |
|  | N | 20 | 20 | 20 | 20 | 20 | 20 |
| CBF value in the L-Calcarine | Pearson Correlation | 0.29 | 0.305 | -0.167 | 0.075 | 0.033 | -0.066 |
|  | Sig. (2-tailed) | 0.215 | 0.191 | 0.482 | 0.753 | 0.891 | 0.782 |
|  | N | 20 | 20 | 20 | 20 | 20 | 20 |
| ** Correlation is significant at the 0.01 level (2-tailed). | | | | | | | |
| * Correlation is significant at the 0.05 level (2-tailed). | | | | | | | |

**Table S4: Pearson correlation between Clusters found in LSSNHL and clinical variables**

| **Brain Region** |  | **BV (low shear) (mPa·s)** | **BV (medium shear) (mPa·s)** | **BV (high shear) (mPa·s)** | **Urine specific gravity (µmol/L )** | **Daily water intake (ml)** | **HHIA** |
| --- | --- | --- | --- | --- | --- | --- | --- |
| CBF value in the L-STG | Pearson Correlation | -0.560** | -0.085 | -0.097 | -0.485* | 0.568** | -0.403 |
|  | Sig. (2-tailed) | 0.007 | 0.706 | 0.669 | 0.022 | 0.006 | 0.063 |
|  | N | 22 | 22 | 22 | 22 | 22 | 22 |
| CBF value in the R-MTG | Pearson Correlation | 0.2 | 0.204 | -0.017 | 0.178 | -0.233 | 0.065 |
|  | Sig. (2-tailed) | 0.373 | 0.364 | 0.94 | 0.427 | 0.296 | 0.775 |
|  | N | 22 | 22 | 22 | 22 | 22 | 22 |
| CBF value in the L-TPSTG | Pearson Correlation | 0.127 | 0.407 | -0.213 | 0.099 | -0.022 | -0.06 |
|  | Sig. (2-tailed) | 0.572 | 0.06 | 0.342 | 0.662 | 0.921 | 0.792 |
|  | N | 22 | 22 | 22 | 22 | 22 | 22 |
| CBF value in the L-Rolandic operculum | Pearson Correlation | -0.065 | -0.419 | 0.042 | -0.055 | 0.135 | -0.018 |
|  | Sig. (2-tailed) | 0.774 | 0.052 | 0.854 | 0.807 | 0.55 | 0.602 |
|  | N | 22 | 22 | 22 | 22 | 22 | 22 |
| ** Correlation is significant at the 0.01 level (2-tailed). | | | | | | | |
| * Correlation is significant at the 0.05 level (2-tailed). | | | | | | | |
